# Immune Checkpoint Modulation in Colorectal Cancer: What's New and What to Expect

**DOI:** 10.1155/2015/158038

**Published:** 2015-10-29

**Authors:** Julie Jacobs, Evelien Smits, Filip Lardon, Patrick Pauwels, Vanessa Deschoolmeester

**Affiliations:** ^1^Center for Oncological Research, University of Antwerp, 2610 Wilrijk, Belgium; ^2^Department of Pathology, Antwerp University Hospital, 2650 Edegem, Belgium; ^3^Laboratory of Experimental Hematology (LEH), Vaccine and Infectious Disease Institute, University of Antwerp, 2650 Edegem, Belgium

## Abstract

Colorectal cancer (CRC), as one of the most prevalent types of cancer worldwide, is still a leading cause of cancer related mortality. There is an urgent need for more efficient therapies in metastatic disease. Immunotherapy, a rapidly expanding field of oncology, is designed to boost the body's natural defenses to fight cancer. Of the many approaches currently under study to improve antitumor immune responses, immune checkpoint inhibition has thus far been proven to be the most effective. This review will outline the treatments that take advantage of our growing understanding of the role of the immune system in cancer, with a particular emphasis on immune checkpoint molecules, involved in CRC pathogenesis.

## 1. Introduction

Colorectal cancer (CRC) is the third most commonly diagnosed cancer worldwide, with almost 1.4 million new cases in 2012 [1]. Patient survival is highly dependent on the tumor stage at the time of diagnosis. Only 40% of CRC cases are diagnosed at an early stage and approximately 50% of recently diagnosed patients will progress to metastatic cancer [2]. The overall 5-year survival of CRC patients is close to 65% ranging from 90% for patients with localized disease to 70% and 13% for patients with localized lymph node metastases or organ metastases, respectively [2]. Although surgery remains the cornerstone in the treatment of this disease, 30–40% of patients have locoregionally advanced or metastatic disease that cannot be cured by surgery alone [3]. Hence, patients at increased risk of disease recurrence and patients with metastatic disease receive adjuvant chemotherapy. Despite the recent progress in diagnosis and treatment, including the introduction of targeted therapies, the prognosis of these advanced CRC remains poor [4].

Advances in molecular biology have helped elucidate some of the genetic mechanisms leading to colorectal carcinogenesis. Most CRC cases are due to sporadic genetic and/or epigenetic changes, but up to 10–20% of all CRC cases have a familial component [2]. There are three major molecular mechanisms that cause aberrant gene expression in CRC: microsatellite instability (MSI), chromosomal instability (CIN), and the CpG island methylator phenotype (CIMP) [2, 5]. Accumulating evidence suggests that tumor progression is governed not only by genetic changes intrinsic to cancer cells but also by environmental factors. Hence, in addition to genetic mutations and TNM staging, a quantitative assessment of immune cells that infiltrate the tumor tissue and peritumoral areas has been proposed as an independent outcome predictor [4]. Increased understanding of the immune tumor microenvironment has allowed for investigation into novel immune-based biomarkers and the development of new agents that target immune pathways for therapy [6]. Among the most promising approaches is the blockade of immune checkpoint molecules to activate antitumor immunity [7]. Therefore, this review will outline the treatments that take advantage of our growing understanding of the role of the immune system in cancer, particularly highlighting immune checkpoint blockade in CRC.

## 2. Antitumor Immunity in CRC

### 2.1. Immune Surveillance and Immunoediting

Through immune surveillance, the body can effectively recognize and eliminate cancerous cells prior to clinical expression [6, 8]. In humans, the role of immune surveillance was first suspected with the observation of increased occurrence of cancer in patients with immunodeficiency. This concept of immune surveillance has long been questioned until it was finally demonstrated in animal models by Shankaran et al. [9]. The selection pressure exerted by the immune system on tumor cells allows resistant clones to escape immune surveillance in a process known as immunoediting [6, 8]. This reciprocal relationship between immune cells and cancer cells occurs in three phases: the immune surveillance period, the latency period, corresponding to a state of equilibrium, and the phase of immune escape, allowing tumor progression and clinical expression [8]. Hence, this complex interaction between tumor cells and the local immune response results in a balance between tumor-promoting and tumor-controlling effects and calls for a close collaboration between cells of the innate immune system and cells of the adaptive immune system [3].

### 2.2. Innate Immunity

Innate immunity is the first line of defense for the antitumor immune system. Innate immune cells have specialized surface receptors that recognize tumor-specific antigens on cancer cells. Recognition initiates an inflammatory cascade leading to antigen presentation by dendritic cells (DCs) and macrophages to T cells, activating an adaptive immune response. Basically, the innate immune system recognizes tumor-specific antigens on the surface of cancer cells in a similar way as the recognition of non-self-pathogens [6].

Natural killer (NK) cells are one of the key cell types involved in immune surveillance [6]. They do not express antigen specific receptors but are able to eliminate neoplastic cells in the absence of certain major histocompatibility complex (MHC) molecules on target cells [3, 10]. In addition, NK cells may exert a cytotoxic effect against cancer cells through other mechanisms such as antibody dependent cell mediated cytotoxicity (ADCC) and secretion of cytokines, including interferon- (IFN-) *γ*, leading to the activation of other inflammatory cells, including macrophages and DCs (see below) [6, 10]. In CRC, an extensive intratumoral infiltration of NK cells has been reported to be associated with a better prognosis [11, 12].

Natural killer T (NKT) cells share characteristics of both T cells and NK cells by coexpressing *αβ* T cell receptor and a variety of molecular markers that are typically associated with NK cells. NKT cells recognize glycolipid antigens like *α*-galactosylceramide presented by CD1d, an MHC class I-like molecule that binds self and foreign (glycol) lipids. When activated, NKT cells secrete abundant proinflammatory cytokines (such as interleukin- (IL-) 2, IFN-*γ*, tumor necrosis factor- (TNF-) *α*, and IL-4) and effector molecules involved in cell death (such as perforin, Fas ligand, and TRAIL). Similar to NK cells, increased tumor infiltration of NKT cells seems to be associated with a better prognosis in CRC [8].

Recruited macrophages and DCs phagocytose tumor cells and can then present tumor-associated antigens (TAAs) on their surface [6]. DCs form a network of antigen-presenting cells (APCs) that shape immune responses by linking innate and adaptive immunity. In order to instruct naïve T cells into the required functional profile, DCs must present tumor antigens via MHC class I and II molecules, express costimulatory ligands, and secrete inflammatory mediators such as IL-12 or type I IFNs [13]. Macrophages at their turn have Fc receptors on their surface and mediate ADCC. Tumor-infiltrating macrophages (TIM) can be divided into two subtypes. M1 TIM secretes high levels of nitric oxide synthase and proinflammatory molecules (IL-6, IL-12, IL-13, and TNF-*α*) and promotes adaptive immunity through increased expression of MHC and costimulatory molecules [8, 10]. In contrast, M2 TIM produces arginase and immunosuppressive cytokines [IL-10 and transforming growth factor- (TGF-) *β* and prostaglandin E2] and promotes angiogenesis via production of vascular endothelial growth factor (VEGF) thereby promoting tumor progression. Tumor-associated macrophage (M2-polarized) infiltration into the tumor microenvironment is generally considered a poor prognostic marker in several tumor types. Conversely, their role in CRC is controversial with numerous studies indicating a better outcome in CRC patients [14–16], while others state the opposite [17].

### 2.3. Adaptive Immunity

Adaptive immunity is responsible for a long-term specific antitumor immune response, including immune memory related to a prior immune challenge [8, 10]. Briefly touching upon adaptive immune cells, B cells play a major role in humoral adaptive immunity and are involved in sustaining long-term immunity [3, 10]. In addition, tumor-infiltrating B cells can sustain and enhance T cell responses by producing antibodies, stimulatory cytokines, and chemokines in addition to functioning as a local APC.

T cells recognize the signaling complex comprised of *αβ* T cell receptor dimer, CD4 or CD8 accessory molecules, and CD3 along with the peptide antigen presented in the context of MHC class I and II molecules [3, 10]. CD4^+^ T cells recognize class II MHC molecules presented on APC, whereas CD8^+^ T cells identify class I MHC molecules expressed on several cell types [10]. Activation of T cells requires 3 signals: recognition of tumor cell antigen, activation of costimulatory molecules (CD80/CD28 and CD40/CD40L), and binding of cytokines (IL-1, IL-2, IL-6, IL-12, and IFN-*γ*) [8]. Upon activation, CD4^+^ T cells can modulate the antitumor immune response. Depending on the cytokine profile produced by the effector cells, CD4^+^ T cells are subdivided in different T helper (Th) subsets, each secreting specific cytokines [3]. Th1 cells secrete cytokines such as IL-2 and IFN-*γ* which promote antitumor immune response by cytotoxic T lymphocytes. In contrast, Th2 cells secrete IL-4, IL-5, and IL-13, promote IgE synthesis, and are believed to favor tumor growth [8, 10]. The most recent addition to effector Th subsets is Th17 cells which develop from naïve CD4^+^ T cells in the presence of TGF-*β*, IL-6, and IL-1*β* and are maintained for a long term in the presence of IL-21 and IL-23. In addition to producing IL-17A, Th17 cells can produce IL-17F, IL-21, IL-22, IFN-*γ*, and granulocyte-macrophage colony-stimulating factor (GM-CSF) [18]. Th17 cells play a complex and controversial role in tumor immunity, either promoting or suppressing tumor growth depending on the malignancy and on the therapeutic intervention investigated. Recent findings also point to significant alterations in Th17 cells due to their interplay with cytotoxic CD8^+^ T cells and regulatory T lymphocytes (Tregs) within the tumor microenvironment [19].

Tregs, of which the best characterized subpopulation expresses CD4, CD25, and Foxp3, function as checkpoint regulators to maintain immune self-tolerance and suppress immune effects against self-antigens [8, 10]. This means that TAAs themselves may induce an increased number of intratumoral Tregs in varying tumor types, including CRC, supporting a role for Tregs in cancer-induced immunosuppression. Hence, targeting Tregs might have an important impact on immunotherapeutic anticancer strategies and the clinical outcome of cancer patients [3]. Activated CD8^+^ T cells can expand clonally and differentiate into “killer lymphocytes” which will recognize and lyse tumor cells using granule exocytosis and formation of FAS ligand. While most cytotoxic T lymphocytes (CTLs) die through apoptosis following effectuation of their killer function, some become long-lived memory cells [3]. Pronounced lymphocyte infiltration has been described in CRC, is more present in MSI tumors, and is reported to be associated with a better clinical course [20]. T cell activation is regulated by a balance between costimulatory and inhibitory signals (i.e., immune checkpoints). Under normal physiological conditions, immune checkpoints are crucial for the maintenance of self-tolerance. Conversely, tumors are capable of deregulating the expression of these immune checkpoint proteins as an important immune resistance mechanism [21].

## 3. Immune Checkpoints in CRC

**Figure 1:**
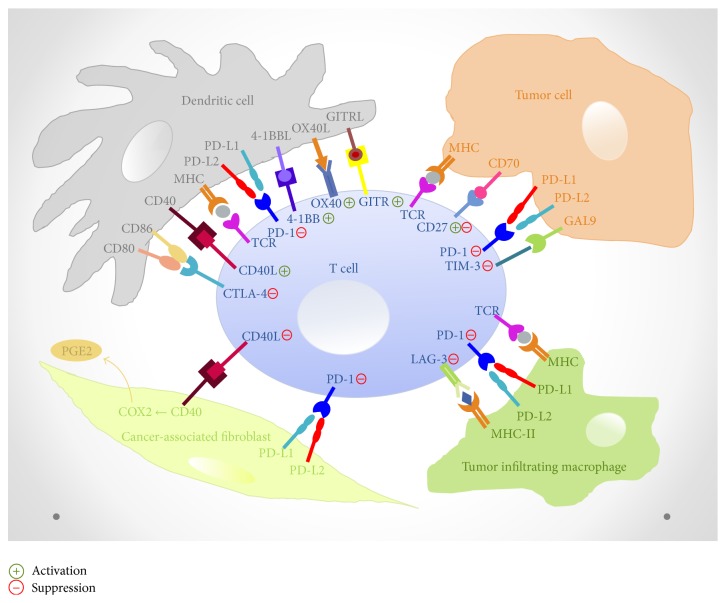
Overview of immune checkpoint molecules involved in CRC pathogenesis. CD, cluster of differentiation; COX2, cyclooxygenase-2; CTLA-4, cytotoxic T lymphocyte antigen-4; GAL9, Galectin-9; GITR, glucocorticoid-induced TNFR-related protein; LAG-3, lymphocyte activation gene-3; MHC, major histocompatibility complex; PD-1, programmed death-1; PD-L, programmed death ligand; PGE2, prostaglandin E2; TCR, T cell receptor; TIM-3, T cell immunoglobulin and mucin containing protein-3.

A major turning point in cancer immunotherapy came with the clinical application of antibodies that block immune checkpoints [22]. Blockade of these inhibitory coreceptors and pathways, which restrain T cell functions in normal physiologic settings and are being exploited by tumors, might “release the brakes” on immune responsiveness leading to tumor elimination [23]. On the other hand, numerous immune checkpoints that enable “stepping on the gas” of immune responsiveness have been identified. In this section we will discuss emerging immune checkpoints in CRC pathogenesis (Figure 1).

### 3.1. PD-1/PD-L1

Programmed death-1 (PD-1, also known as CD279) is a coinhibitory receptor that is inducibly expressed on CD4^+^ T cells, CD8^+^ T cells, NKT cells, B cells, and monocytes/macrophages [24]. Known ligands of PD-1 include PD-L1 (B7-H1) and PD-L2 (B7-DC). PD-L1 is constitutively expressed on a wide variety of immune cells and nonimmune cells, whereas for PD-L2 this expression can be induced, depending on microenvironmental stimuli [25]. This pathway has been implicated in tumor immune evasion due to the upregulation of PD-1 on tumor-infiltrating lymphocytes (TILs) and increased expression of its ligands on tumor cells, leading towards suppression of tumor-specific CD8^+^ T cells. Furthermore, this pathway has been associated with T cell exhaustion in cancer as defined by impairment of proliferation, cytokine production, and cytotoxicity [26]. To overcome this immune suppression, blocking monoclonal antibodies (mAbs) against PD-1 and PD-L1 are emerging and have shown durable responses in metastatic solid tumors.

A role for this pathway in CRC pathogenesis was first shown by the correlation of single nucleotide polymorphisms in the PD-1 gene with CRC in a Chinese population [27] as well as with colon cancer in Iranians [28]. Thereafter, PD-1 was shown to be markedly upregulated on CD8^+^ T cells in the tumor microenvironment of CRC specimens in comparison to CD8^+^ tumor-free lymph nodes. Moreover, these PD1^+^CD8^+^ T cells in the tumor microenvironment were associated with the impairment of cytokine and perforin production [26]. Interestingly, the expression level of PD-L1 on CRC seemed to be the crucial player in this impairment of cytokine production [26]. In addition, using immunohistochemistry (IHC), Hua et al. revealed an inverse relationship between the expression of PD-L1 on CRC cells and T cell density in the tumor microenvironment [29]. Next to the reduction in T cells, an expansion of Tregs could be found, marked by the high number of Foxp3^+^ cells and a strong correlation between PDL-1^+^ tumor cells and worse prognosis [29]. Also, in peripheral blood from postsurgical CRC patients, PD-1 expression could be demonstrated on both CD4^+^ and CD8^+^ T cells and again marked impaired T cell function [30]. Based on these data, the blockade of PD-1/PD-L1 interaction has been proposed as a therapeutic strategy in CRC. Unfortunately, in a clinical setting, no objective clinical responses of anti PD-1 therapy (BMS936558/Nivolumab/MDX-1106) were observed in 19 CRC patients [31]. Also, in 2012, no response to therapy was seen in 18 CRC patients, using an antagonistic PD-L1 antibody (BMS936559/MDX-1105) [32]. Furthermore, a study by Droeser et al. demonstrated an association of PD-L1 expression with improved survival in CRC specimens [33]. Moreover, a significant correlation between PD-L1 overexpression, infiltration of PD-1^−^CD8^+^ lymphocytes, and IFN-*γ* gene expression was observed. Remarkably, this correlation could only be demonstrated in a subset of CRC patients, marked by mismatch repair (MMR) proficient tumors, whereas no association was found in MMR-deficient CRC, also known as MSI [34]. The idea that immune checkpoint blockade could be more effective in MSI CRC was further investigated by a small phase 2 trial of Pembrolizumab, another fully human mAb targeting PD-1. Indeed, this study showed that MMR status predicted clinical benefit of immune checkpoint blockade with Pembrolizumab, with enhanced responsiveness in MSI CRC [35]. In addition, Nivolumab (MDX-1106) was tested in patients with advanced treatment-refractory solid tumors, including 14 CRC patients of which one patient achieved a complete response (CR) with no evidence of disease recurrence after three years [23]. Likewise, further studies on the tumor of this patient demonstrated microsatellite instability [36]. The importance of the MMR status for response to therapy is getting more and more clear, demonstrated by the multiple active clinical trials with anti-PD-1 (AMP-224, PDR001, Nivolumab, Pembrolizumab, REGN2810, BGB-A317, and MEDI0680) and anti-PD-L1 therapy (MEDI4736, MDX-1105, Avelumab, and MPD-L3280A) enrolling more patients with MSI status (Table 1). Next to the growing landscape of mAbs, targeting the PD-1/PD-L1/L2 axis in monotherapy, different combination strategies are emerging in these trials using other immune checkpoint inhibitors (Ipilimumab and MEDI4736), immunostimulatory molecules (Denenicokin, RO6895882, Lirilumab, and PF-05082566), targeted therapies (Cobimetinib and Avastin), or conventional therapies (stereotactic body radiation, hypofractionated radiotherapy, and cyclophosphamide) (Table 1).

### 3.2. CTLA-4/B7

Another molecule involved in T lymphocyte inhibition is cytotoxic T lymphocyte antigen-4 (CTLA-4), expressed on the surface of T lymphocytes. CTLA-4 has similar binding affinities for B7-1 (CD80) and B7-2 (CD86) costimulatory receptors on APC and this interaction transmits inhibitory signals to attenuate T cell activation by competing for B7 ligands with its homologue, CD28 [24]. Therefore, CTLA-4 is an interesting target to block with monoclonal antibodies. One such example is Ipilimumab, currently FDA approved for first-line and second-line treatment of metastatic malignant melanoma. Here, Ipilimumab has been shown to reinvigorate the antitumor immune response by binding CTLA-4 and thereby preventing it from binding to its ligands and reducing the inhibition of CD28/B7 T cell activation. Next to the inhibition of T cell activation, this also resulted in the reduction of Tregs. Since Treg accumulation has been linked with poor outcome in CRC, this might be an interesting therapeutic strategy for CRC [37].

Similar to PD-1, a role of CTLA-4 in CRC development was suggested by multiple groups, showing associations of CTLA-4 single nucleotide polymorphisms and the risk of developing CRC [27, 38–40]. CTLA-4 49A/G polymorphism came forth as a major player in CRC development. It was also demonstrated that CTLA-4 is expressed at considerably higher levels in MSI tumors as compared to MSS [41]. Here, the expression of CTLA-4 was found not only on TILs intercalated within the epithelial component of the tumor but also in the surrounding tumor stroma and at the invasive front of the tumor. Of particular interest is also the expression of CTLA-4 on multiple subsets of Tregs. First, a significant increase of activated Tregs (CD45RA^−^Foxp3^+^ T cells) in peripheral blood and cancer tissue of patients with colon cancer was demonstrated with significantly higher levels of CTLA-4 [42]. Second, accumulation of CCR4^+^CTLA-4^+^ regulatory T cells was found in colon adenocarcinomas as well as an increase in CTLA-4^+^ conventional T cells, susceptible to immune regulation in the tumor-associated mucosa [43]. Finally, the presence of a potent suppressive CD4^+^Foxp3^−^ T cell population was revealed within the colorectal tumor regulatory landscape by comparison of healthy colon, colorectal tumor samples, and matched blood from CRC patients [44]. These CD4^+^Foxp3^−^ T cells seemed to coexpress immune checkpoints such as LAG-3, PD-1, and CTLA-4 and were able to produce immunosuppressive cytokines such as IL-10 and TGF-*β*. More importantly, this unique population was 50-fold more suppressive than Foxp3^+^ Tregs. The expression of CTLA-4 on different subsets of regulatory T cells makes this immune checkpoint an interesting therapeutic strategy, which might lead to strengthening of the antitumor immune response in CRC [44]. In this regard, Tremelimumab, a similar antibody to Ipilimumab, has been investigated in a phase II study for patients with refractory metastatic adenocarcinoma of the colon or rectum who failed standard chemotherapy. Surprisingly, only a single patient received a second dose, whereas the remaining 46 patients had disease progression or disease-related death before reaching the planned second dose at 3 months [45]. Because these data do not support further investigation of Tremelimumab as a single agent for the treatment of advanced, treatment-refractory colorectal cancer, phase I trials are now ongoing in combination with MEDI4736, a PD-L1 antagonistic mAb, in patients with solid tumors. Furthermore, phases I and I/II of Ipilimumab are actively recruiting patients with metastatic solid tumors in combination with stereotactic body radiation or Lenalidomide (Table 1).

### 3.3. TIM-3

T cell immunoglobulin and mucin containing protein-3 (TIM-3) was discovered as a molecule expressed on IFN-*γ* producing CD4^+^ Th1 and CD8^+^ cytotoxic T cells. Through its ligand, Galectin-9, TIM-3 is believed to play a critical role in inhibiting Th1 responses and inducing cell death [46]. Furthermore, animal models have revealed its role in T cell exhaustion due to the expression of TIM-3, together with PD-1, in the most suppressed or dysfunctional populations of CD8^+^ T cells in hematological as well as solid malignancies. In preclinical models, blocking TIM-3 was able to reinvigorate antitumor activity, comparable to the effect of PD-1 blockade with a greater efficacy through combination of both.

In peripheral blood samples from CRC patients, Xu et al. demonstrated significantly higher levels of circulating TIM-3^+^PD-1^+^CD8^+^ T cells compared to healthy blood [47]. Also peripheral blood, drawn after surgery, exposed the expression of TIM-3 and PD-1 on CD8^+^ T cells and CD4^+^ T cells. Moreover, the expression of both TIM-3 and PD-1 appeared to relate with the impaired function of these T cells [30]. Likewise, an increase of Tim-3^+^PD-1^+^CD8^+^ T cells was observed in CRC tissue, when compared to tissues adjacent to the tumor. Interestingly, distinguishing the subset of T cells by the expression of PD-1 demonstrated a significant lower level of IFN-*γ* production in the PD-1^−^ subset. Together with the lack of objective responses by PD-1 blockade in a large population of CRC patients (as discussed above), these results suggest TIM-3 as a more dominant inhibitory receptor, restricting T cell responses in CRC patients. In addition, blocking this pathway might restore the impaired cell-mediated immunity after surgical resection. Taken together, these data support the development of TIM-3 inhibitors and hold great promise as single or combined modalities in CRC patients [48].

### 3.4. LAG-3

Another interesting target for immune checkpoint blockade is lymphocyte activation gene-3 (LAG-3, also known as CD223), a cell surface molecule of the immunoglobulin superfamily. Through its interaction with MHC class II, LAG-3 has been demonstrated to play a pivotal role in negative regulation of T cell proliferation, enabled by its expression on activated T cells, NK cells, B cells, and plasmacytoid DCs [48, 49]. In addition, LAG-3 appears to mitigate Treg function. Indeed, the expression of LAG-3 on CD4^+^CD25^+^ cells was able to define a subset of cells endowed with potent suppressor activity [50]. Together with CD49b, the expression of LAG-2 marks highly suppressive human type 1 regulatory T cells (Tr1), a subgroup of Tregs producing IL-10 [51]. It was also recently revealed that exhausted CD8^+^ T cells can express LAG-3 and that the expression of multiple inhibitory receptors, such as the combination with PD-1, was associated with greater T cell exhaustion. Moreover, simultaneous inhibition of PD-1 and LAG-3 could enhance T effector activity as compared to either molecule alone [52]. Henceforward, clinical trials with LAG-3 inhibitors (LAG-525 and BMS-986016) are now progressing into phase I studies, with or without the combination of PD-1 inhibitors (Nivolumab and PDR001) in patients with advanced solid malignancies (Table 1).

J. Chen and Z. Chen examined 108 CRC tissues and their healthy colorectal mucosa and demonstrated a significant increase in the percentage of LAG-3^+^/CD49b^+^ cells as compared with peritumoral tissues [53]. The increase of Tr1 cells in tumor tissues suggests a crucial role for this subset of cells in CRC progression and seems to be predictive for poor prognosis. It is therefore not unexpected that clinical trials with LAG-3 inhibitors have been designed to enroll CRC patients (Table 1).

### 3.5. CD70/CD27

Although expression of CD70, a member of the tumor necrosis factor family, is normally restricted to activated T and B cells and mature dendritic cells, constitutive expression of CD70 in tumor cells has been described [54]. Through its ligand, CD27, the upregulation of CD70 by tumor cells can facilitate evasion of the immune system by three important mechanisms: induction of T cell apoptosis, skewing T cells towards T cell exhaustion, and increasing the amount of suppressive Tregs [55]. Moreover,* in vivo* experiments demonstrated evasion of immune surveillance by recruitment of CD27^+^ Treg to the tumor site [56]. The role of CD70-mediated immune escape was also demonstrated in non-small cell lung cancer (NSCLC), where CD27^+^ lymphocytes were found in the tumor microenvironment with a trend towards increased Foxp3 expression and higher CD4/CD8 ratios surrounding CD70^+^ tumor cells [57]. Although expression of CD70 in tumor cells of colorectal origin has not been published to date, preliminary data of our group showed expression of CD70 in 6/28 CRC biopsies (Jacobs et al., unpublished data). Furthermore, immunohistochemistry on colon biopsies revealed expression of CD70 in 9% of cases (17/194) [58].

These observations, paired with the limited expression profile of CD70 in normal conditions, present an interesting opportunity to target this molecule in CRC. To date, three anti-CD70 immunoglobulins have entered clinical trial of which ARGX-110 is the only one, enrolling solid and hematological CD70^+^ advanced malignancies in the study (Table 1).

Contrary to the CD70-blocking strategy, other groups focus on the immunostimulatory potential of a CD27-agonistic monoclonal antibody, such as Varlilumab (Table 1). CD27 belongs to the tumor necrosis factor receptor superfamily (TNFRSF) and plays a key role in immunological processes, such as T cell survival, T cell activation, and the cytotoxic activity of NK cells [59]. Furthermore, ligation of CD27 by CD70 has shown stimulatory effects on T cell proliferation, expansion, and survival dependent upon IL-2 autocrine signaling [60, 61]. As stated above, CD27 triggering may also lead to tumor progression through the recruitment of CD27^+^ Tregs, complicating the use of CD27 as a target for immunotherapy. However, a fully human monoclonal CD27 agonist antibody, CDX-1127, is being evaluated in solid malignancies, with or without the administration of Nivolumab (see Table 1) and seems able to tear apart the inhibitory and costimulatory mechanisms [60]. Moreover, tumor shrinkage in one CRC patient has already been demonstrated in the dose escalation study [62].

### 3.6. OX40 (CD134)

OX40, also known as CD134, is another costimulatory immune checkpoint molecule of the TNFRSF, capable of stimulating therapeutic immune responses. This molecule has been shown to be transiently upregulated on CD4^+^ and CD8^+^ T cells after T cell receptor engagement and during antigen specific priming [63]. Its ligand, OX40L, is presented on activated antigen-presenting cells as well as activated endothelial cells, epithelial cells, and B cells [64]. Furthermore, nonclinical models show OX40 cell surface expression is induced following activation of NK cells with enhanced NK cell activity upon ligation of OX40. Preclinical studies with anti-OX40 agonistic mAb show augmented T cell differentiation, survival, expansion, and cytolytic function [65]. In addition to promoting effector T cell expansion, OX40 agonists have the ability to directly regulate Treg cells by diminishing its inhibitory effects and thereby promoting antitumor CD8^+^ T cell responses necessary to maintain long-term antitumor immune responses [63].

OX40^+^CD4^+^ TILs have been detected in breast cancer, sarcoma, and melanoma as well as CRC. Indeed, Petty et al. demonstrated high levels of OX40^+^ lymphocytes in half of primary CRC specimens with a significant correlation towards better survival in the latter [65]. Moreover, OX40 expression levels were the highest inside the tumor and significantly decreased towards the direction of the tumor border and healthy tissue in 39 CRC patients [64]. These results suggest a weakened immune response at the border of the tumor, making it an interesting target for immunotherapy in CRC.

*In vivo* OX40 agonistic antibodies showed regression of at least 1 metastatic lesion in 12 out of 30 patients after only 1 cycle of treatment [66]. Despite these positive results, it is unlikely that anti-OX40 alone will be sufficient to induce complete response, since antitumor immunity is directed by a dynamic constellation of signals. Therefore, maximizing the therapeutic benefit of OX40 agonists (MEDI6469, MEDI6383, and MOXR0916) will likely depend on the combination with antagonistic Abs, like PD-L1 (MEDI4736 and MPL3280A) and CTLA-4 (Tremelimumab) targeting antibodies (see Table 1) [63].

### 3.7. GITR

Glucocorticoid-induced TNFR-related protein (GITR, also known as CD357) is a surface receptor molecule that has been shown to be involved in inhibiting the suppressive activity of Tregs and extending the survival of T effector cells. Therefore, it may hold great promise for the generation of agonistic antibodies. Next to the transient expression on activated CD4^+^ and CD8^+^ T cells and the constitutive expression on Tregs, expression has been observed on DC, monocytes, and NK cells [60]. GITRL, its unique ligand, is highly expressed on activated APCs and endothelial cells and ligation with GITR appears to provide costimulation of effector T lymphocytes [67]. Preclinical studies have shown that GITR agonistic agents (like DTA-1) can mediate tumor regression in animal models in part based on a unique mechanism causing Tregs to lose lineage stability, reducing their suppressive influence over the tumor microenvironment [68]. Furthermore, T cell stimulation through GITR attenuates Treg-mediated suppression or enhances tumor-killing by CD4^+^ and CD8^+^ effector T cells. Furthermore, a synergistic effect was shown after coadministration of anti-GITR with adoptive T cell transfer and anti-CTLA-4 mAbs, leading to eradication of more advanced tumors [60, 69, 70]. This latter effect was confirmed in murine models bearing fibrosarcoma or CRC.

In CRC patients with liver metastasis, the tumor-specific T cell response is comprised by high numbers of activated Tregs, expressing high levels of GITR and inducible T cell costimulator (ICOS) [67]. Moreover, treatment with soluble GITRL was able to inhibit Treg-mediated suppression, preventing hyporesponsiveness of effector T cells [67]. Although to date preclinical data supporting the use of agonistic GITR mAb for immunotherapeutic interventions in CRC are scarce, two GITR agonistic antibodies (TRX518 and MK-4166) are being investigated in a phase I setting, with or without the addition of a PD-1 inhibitor (Pembrolizumab) (Table 1).

### 3.8. 4-1BB (CD137)

4-1BB, also known as CD137, is a member of the TNFRSF and is widely known as a T cell costimulatory receptor induced after T cell antigen recognition. 4-1BB binds a high-affinity ligand, 4-1BBL, present on APCs to transduce signals for T cell growth and differentiation. Although both CD4^+^ and CD8^+^ T cells express 4-1BB at similar levels, upon activation, signals through 4-1BB are more biased towards CD8^+^ T cells [71]. Besides its expression on T cells, 4-1BB is expressed, albeit at low levels, on a multitude of cells of the hematopoietic lineage including B cells, regulatory T cells, NKs, NKTs, DCs, mast cells, and early myeloid progenitor cells [72]. Also, a number of studies showed expression of 4-1BB on a wide range of tumor cells [71, 72]. The broad range of 4-1BB expression on multiple cell types makes this receptor a dual-edged sword in the fight against cancer as 4-1BB agonists might elicit strong antitumor responses from a myriad of cell types, however, sometimes at the cost of off-target immune pathology [72].

Cepowicz et al. studied the expression of 4-1BB in peripheral blood samples of 72 patients with primary CRC and demonstrated a direct correlation of 4-1BB positivity and CRC stage as well as invasion depth [73]. Furthermore, an increase in 4-1BB (as well as CD134) was found in peripheral blood taken after surgical resection for CRC, which might be due to increased IL production after elimination of a tumor. On the other hand, expression of its ligand was shown to be lower in cancerous colon tissue compared with paired normal tissue [74]. Suppressed levels of 4-1BBL might indicate the involvement of this pathway in immune escape of colon tumors by the decreased interactions of T cells with tumor cells and macrophages. Interestingly, patients harboring this increased expression of 4-1BB were shown to have high soluble 4-1BB levels in their plasma [74]. Interaction of this soluble 4-1BB with 4-1BBL has been shown to control T cell function by inhibiting the ligation of 4-1BBL with 4-1BB and therefore these results suggest a possible feedback loop to reduce further activation of T cells [75]. Interestingly, this could not be shown in rectal cancerous tissue pointing towards different carcinogenesis of CRC based on the tumor location. Furthermore, the beneficial effects of 4-1BB agonism for the treatment of CRC with hepatic metastases have already been demonstrated in animal models [76, 77].

Currently, two 4-1BB agnostic antibodies (Urelumab and PF-05082566) have entered the clinical setting, enrolling patients with advanced solid tumors or B cell non-Hodgkin lymphoma. For Urelumab, dose escalation data revealed an acceptable toxicity rate across a wide dose range (0.3–15 mg/kg) with increasing percentages of circulating activated CD8^+^ and CD4^+^ T cells following a single treatment [78]. Based on these promising results, a phase II study was designed in patients with metastatic melanoma. Surprisingly, this study was terminated due to fatal hepatotoxicity. Henceforward, further trials are mainly focused on low-dose therapies in combination with approved mAbs (Table 1) [79]. For PF-05082566, no significant elevations in liver enzymes and no dose-limiting toxicities have occurred to date [80]. Moreover, PF-05082566 was well tolerated in a first clinical setting with stable disease, observed in 6 out of 27 patients treated. Although these agonistic antibodies hold promise in monotherapy, an interesting combination strategy of 4-1BB agonistic mAb with monoclonal antibodies equipped to induce ADCC was shown [81]. Of interest here is Cetuximab, a human mouse chimeric IgG1 mAb used to treat EGFR expressing* RAS* wild-type metastatic CRC patients. 4-1BB is upregulated on human NK cells when they encounter antibody-bound tumor cells. Moreover, increased levels of 4-1BB on circulating and intratumoral NK cells were directly correlated to an increase in EGFR-specific CD8^+^ T cells and the combination with Cetuximab marked clear synergism, shown by the complete tumor resolution and prolonged survival [81, 82]. Also* in vivo* this combination regimen has been launched in a clinical setting, combining Urelumab (4-1BB) with Cetuximab in CRC and head and neck cancer patients. Additionally, the combination of PF-05082566 with Mogamulizumab, another ADCC-mediating antibody targeting CCR4, has been initiated (Table 1).

### 3.9. CD40

CD40, a final member of the TNFRSF, was initially characterized on B cells and is also expressed on DCs, monocytes, platelets, and macrophages as well as by nonhematopoietic cells such as myofibroblasts, fibroblasts, epithelial, and endothelial cells. The ligand of CD40, known as CD154 or CD40L, is expressed primarily by activated T cells as well as activated B cells and platelets [83]. CD40/CD40L interactions on activated Th cells enhance antigen presentation and expression of costimulatory molecules, licensing DC to mature and achieve all of the necessary characteristics to effectively trigger T cell activation. In murine models, engagement by CD40L promoted cytokine production and enabled effective T cell activation and differentiation [84]. Except for its expression in cells of hematopoietic origin, expression of CD40 has also been demonstrated in several types of carcinoma cells, rendering them susceptible for apoptosis [85]. Interestingly CD40 expression seems absent on normal epithelium, suggesting that expression confers a growth advantage in early stages of malignant development [86]. It has been suggested that neoplastic growth utilizes the CD40/CD40L pathway independent of the immune system to sustain proliferative capacity and survival. Moreover, this receptor/ligand interaction enables tumors to manipulate both T cell and APC compartments most likely contributing to the establishment of the immunosuppressive tumor microenvironment [83].

In CRC, Georgopoulos et al. were the first to demonstrate expression of CD40 in CRC cell lines and colon cancer, with strong (2/17), moderate (4/17), or weak (11/17) positivity in the tumor cells [87]. CD40L was also detected in a number of primary colorectal carcinomas, suggesting an important role of CD40/CD40L axis in CRC tumor immunity (Baxendale et al., unpublished observations) [86]. Contrary to the importance of CD40 expression in early stages of malignant development, progression of malignancy renders cells susceptible to direct antiproliferative effects and CD40-mediated growth inhibition or apoptosis, leading to loss of CD40 expression [88]. Consequently, the use of CD40 as a prognostic tool has been demonstrated, although further research to elucidate its role in CRC is mandatory [87, 89]. Nevertheless, CD40^+^ TAM and plasma sCD40 in colorectal cancer tissues have already been found to be favorable prognostic markers [90]. In addition, using membrane bound CD40L, but not soluble agonists, a powerful proapoptotic signal and proinflammatory cytokine production could be triggered in CRC cells [87]. These results suggest that CD40 is a promising therapeutic target for the eradication of CRC tumors.

Preclinical investigations with CD40 agonists have been robust and highlight multiple mechanisms of action to overcome tolerance and drive potent T cell immunity in lymphoma and certain solid tumors. Initial clinical trials of agonistic CD40 mAb have shown clinical activity in the absence of disabling toxicity. However, overall response rates remain 20% or less, proposing that CD40 agonists will be most effectively used in combination with other modalities such as chemotherapy, radiation, and vaccines or with negative checkpoint molecule blockers like anti-CTLA-4 or anti-PD-L1 mAbs [91]. In this regard, the safety of CP-870, 893, a fully human CD40 agonistic mAb with carboplatin and paclitaxel, was assessed in a phase I study. Of the 30 evaluable patients, 6 exhibited partial responses, providing a rationale for phase II studies [92]. To date, four other CD40-agonistic antibodies (ADC-1013, RO7009789, SEA-CD40, and ChiLob 7/4) are enrolled in a phase 1 clinical setting with or without the combination of PD-L1 blocking antibodies (MPD-L3280A).

## 4. Role of Cancer Associated Fibroblasts in Immunomodulation

Increasing evidence has suggested that antitumor efficacy of cancer immunotherapies could be limited by the presence of cancer associated fibroblasts (CAFs). In CRC, CAFs are the main cellular components of the tumor reactive stroma and play a key role in CRC development enabling the induction of immunosuppressive factors, modulation of the microenvironment to a Th2 phenotype, and inhibition of antigen-specific T cell responses and have been considered the main determinants in metastatic progression [93, 94]. A variety of immune cells aid in this process; for example, monocytes differentiate into a distinct M2 polarized macrophage with poor antigen-presenting capacity and further suppress Th1-adaptive immune responses. Additionally, CAFs are also the principal cells producing extracellular matrix within tumor tissue, providing a physical barrier for the immune attack induced by immunotherapies [93, 95]. Furthermore, CAFs seem able to inhibit the proliferation of activated T cells. Herein, the role of immune checkpoints molecules is becoming more and more clear, such as the expression of PD-L2 on human colonic fibroblasts, resulting in T cell suppression in the gut epithelial mucosa. Of special importance to the field of tumor immunology is the finding that not only normal fibroblasts but also cancer associated fibroblasts can constitutively express PD-L2 [25]. Strikingly, Nazareth et al. and colleagues found constitutively high PD-L1 and 2 expression in fibroblasts that were cultured from human NSCLC [96]. Moreover, this expression appeared to be functional, since* in vitro* blocking studies demonstrated that the fibroblasts inhibited IFN-*γ* production in a PD-L1 and 2 dependent matter. The CD40-CD40L axis appears to be another critical pathway for fibroblast and immune system interaction [97]. Indeed, expression of CD40 has been demonstrated on fibroblasts from human lung, orbit, thyroid, and gingiva. Moreover, during inflammation and in fibrotic conditions, activated T cells, eosinophils, and mast cells displaying CD40 ligand are translocated to sites adjacent to fibroblasts enhancing the inflammatory process by inducing synthesis of cytokine mediators and adhesion molecules [97]. Thereby, activation of the transcription factor NF-kB has been shown, resulting in the secretion of high levels of IL-6 and IL-8 as well as the induction of proinflammatory prostaglandin E2 (PGE2) synthesis by fibroblasts (Figure 1). Interestingly, PGE2 severely inhibits both the acquisition of activating receptors and the release of cytotoxic granules by NK cells, resulting in immune evasion [48]. In contrast to the immunostimulatory potential of an agonistic CD40 mAb, stimulating the CD40/CD40L axis on fibroblast might have detrimental effects on the antitumor response. Therefore, future studies should not merely focus on the expression of immune checkpoint molecules by tumor cells but also take the tumor stroma into account, with a particular focus on CAFs.

## 5. Role of Known CRC Biomarkers in Immunomodulation

Despite recent development and implementation of personalized cancer medicine based on genetic profiling of individual tumors, patient selection for CRC therapy remains challenging. Lately, there has been an increasing interest in biomarkers to predict future patterns of CRC disease. Several promising candidate markers have been investigated for targeted therapies in CRC, including MSI,* KRAS*, and* BRAF* mutations. Furthermore, Galon et al. reported that the adaptive immune response influences the behavior of human tumors [98]. However, the factors that determine a patient's immune phenotype are unclear, and few systematic analyses have investigated the somatic and germline molecular drivers of immune infiltration [99]. Nevertheless, identification of genetic factors that influence the tumor microenvironment is essential to improve the effectiveness of stratified immunotherapy approaches [99].

### 5.1. MSI

Lal et al. carried out a bioinformatic analysis of CRC data in The Cancer Genome Project involving two-dimensional hierarchical clustering to define an immune signature [99]. A group of 28 tightly coregulated immune-related genes were identified and termed the Coordinate Immune Response Cluster (CIRC). An important feature of the CIRC signature is that it includes essentially all class II MHC loci, as well as CD4, whereas, in contrast, expressions of class I MHC molecules, CD8B, and granzyme B are all excluded. In addition, CIRC also included the major immune checkpoint molecules, including PD-L1, PD-L2, LAG-3, TIM-3, and CTLA-4. One of the key aims of this study was to examine the somatic factors associated with the immune response in CRC. It was shown that MSI-high (H), which is the molecular fingerprint of a deficient DNA mismatch repair system and linked to a high mutational burden, is associated with a high immune infiltration characterized by Th cells and class II related genes, ranges of chemokines, and immune inhibitory checkpoint molecules. Hence, MSI-H tumors may be particularly amenable to CD4^+^ cell expansion and adoptive transfer approaches, yet the coordinated expression of checkpoint inhibitor genes observed suggests combination checkpoint blockade therapy may be required to improve efficacy. Similarly, POL (polymerase) mutant tumors, which also have a high mutational burden, were also associated with high CIRC expression.

Likewise, Llosa and colleagues examined the immune microenvironment of primary CRC using IHC, laser capture microdissection/qRT-PCR, flow cytometry, and functional analysis of tumor-infiltrating lymphocytes [41]. It was suggested that MSI represents a classical example of adaptive resistance in which an active immune Th1/CTL microenvironment results in a compensatory induction of checkpoints, including PD-1, PD-L1, CTLA-4, IDO, and LAG-3, which protect the tumor from apoptosis [21, 41]. However, the interface between MSI tumors and T cells seems to be characterized by little expression of PD-L1 on tumor cells despite IFN-*γ* expression by the T cells. Instead, the T cells infiltrate was interlaced with an abundant PD-L1 positive myeloid cell population that presumably inhibits the T cell response. On the basis of these findings, two clinical trials have been initiated to test PD-1 blockade in patients with MSI-H CRC (see Table 1). Combinations with IDO, LAG-3, CTLA-4, and other checkpoints will likely follow [100].

### 5.2. *KRAS*

In contrast to the results on MSI, Lal and colleagues showed that* RAS* mutation predicts for a relatively poor immune infiltration and low inhibitory molecule expression.* KRAS* and* NRAS* mutant CRC had significantly lowered levels of CD4^+^ T cells [99]. Thus, any immunology-based therapy in* RAS* mutant tumors should take into account this immunologically relatively quiescent status of the tumor microenvironment. In this setting, checkpoint blockade may be less efficacious, highlighting the requirement for novel strategies in this patient group [99]. In addition, Kocián et al. examined the correlations between the* KRAS* mutational status, patterns of tumor-infiltrating immune cells, and the presence of tumor recurrence in a cohort of newly diagnosed CRC patients [4]. They observed a significantly higher proliferation rate in tumors with codon 13 mutations as well as a marked variability in the pattern of tumor-infiltrating immune cells regardless of the mutation type. These patients showed a low level of TILs and a high CD1a^+^/CD-LAMP^+^ tumor-infiltrating DC ratio indicating a high risk of cancer-related death. Because the quantification of immune responses within the tumors indicated a strong predictive role in CRC patients, the combined characterization of genetic features and immune cells might provide the foundation to identify high-risk patients [4].

### 5.3. *BRAF*

Finally, activating mutations in* BRAF *have been reported in 5%–15% of CRC cases and are frequently found in MSI-H tumors. While* BRAF *mutation is associated with worse survival in MSS tumors, its role in MSI-H tumors is more controversial. It has been postulated that it is not the* BRAF* mutation itself that confers a poor prognosis but rather the fact that the mutation has different effects depending on the type of genetic pathway in which it is produced [3]. Currently, no data are available on the impact of* BRAF* mutation on the tumor immune landscape of CRC. However, recent evidence indicates that melanomas bearing mutant* BRAF* may also have altered immune responses, suggesting additional avenues for treatment of this patient group [101]. Significant advances in the treatment of melanoma have been made with* BRAF*-targeted therapy, not only leading to significant but short-lived clinical responses in a portion of patients but also leading to immunostimulatory bystander events, which then subside with the emergence of resistance [102]. Combination of* BRAF* inhibitors with new immunotherapies such as checkpoint blockade antibodies might further enhance immune activation or counteract immunosuppressive signals.

## 6. Role of Immunologic Markers

A major turning point in cancer immunotherapy came with the clinical application of antibodies that block immune checkpoints. Hence, the need for clinically useful biomarkers to determine the best way to incorporate these new agents into treatment algorithms for patients with specific diseases is clear [103].

In colon cancer, T cell infiltrates in the primary tumor represent the strongest prognostic parameter compared to the currently used stage-defining parameters [98]. However, such immunological parameters have not routinely been used in clinical practice yet. In addition, determining which patients benefit from immune checkpoint inhibition remains a principal clinical question.

The importance of tumor expression of PD-L1 as a predictive biomarker has been studied extensively, and while tumor expression of PD-L1 can effectively enrich cohorts of patients, it is not a binary predictive marker [104]. Although currently one commercially available PD-L1 antibody (clone E1L3N) has been validated for IHC, the utilization of this antibody for predicting response to anti-PD-1 or anti-PD-L1 therapies remains unknown [105]. Emerging data suggest that patients whose tumors overexpress PD-L1 by IHC have improved clinical outcomes with anti-PD-1-directed therapy, but the presence of patients with PD-L1 negative tumors that also show a robust response complicates the issue of PD-L1 as an exclusionary predictive biomarker [104]. The use of PD-L1 IHC as a predictive marker is confounded by multiple unresolved issues including variable detection antibodies, differing IHC cutoffs, tissue preparation, processing variability, primary versus metastatic biopsies, oncogenic versus induced PD-L1 expression, and staining of tumor versus immune cells [106]. The utility of measuring other inhibitory components of the PD-1/PD-L1 axis such as PD-1 and PD-L2 or the role of immunostimulatory molecules like OX40 is still poorly understood. It is clear that much more information must be gathered not only on the PD-1/PD-L1 axis but also on TILs and other inhibitory/stimulatory pathways to fully understand responses and primary or acquired resistance to immunotherapy. In conclusion, a multitude of questions remain unanswered and need to be resolved to integrate predictive markers for anti-PD-1/anti-PD-L1 therapies into the clinical diagnostic routine [103, 105].

## 7. Discussion

The FDA approval of anti-CTLA-4 for the treatment of metastatic melanoma and of anti-PD-1 for metastatic melanoma and non-small cell lung cancer has engendered new-found awareness among oncologists of the potential antitumor activity of immune checkpoint modulation. In addition, remarkable efficacy of these drugs was shown in renal cell cancer, ovarian cancer, and Hodgkin's lymphoma, even upon failure to several lines of therapy. Despite clinical successes in a diverse range of malignancies, evidences of durable responses in CRC are scarce and appear restricted to MMR-deficient CRC, with its high mutational burden.

In CRC, due to its complicated and close relationship between the stroma and tumor cells, the combination of two or more therapeutic agents might be more effective than merely targeting a single factor. In this regard, abolishing the suppressive factors in the tumor microenvironment is only one step in this cancer-immunity cycle and still requires elimination of cancer by activated T cells. Therefore, another interesting approach could be to not only overcome immunosuppression but also combine this with agonistic antibodies such as GITR, CD27, CD40, 4-1BB, or OX40 to achieve maximum activity of the antitumor response. On the other hand, also the combination of immunotherapy with targeted therapeutics, such as the synergism between 4-1BB agonistic antibodies and Cetuximab, is promising. Nevertheless, preclinical data of combination regimens in CRC is limited and still necessitates the determination of appropriate dosing and treatment schedules of these agents. Finally, well-established biomarker candidates and detection techniques need to be developed along with therapeutic strategies targeting CAFs and the other components in the tumor microenvironment in order to be able to enhance effectiveness of immune checkpoint modulation.

## Figures and Tables

**Table 1:** Clinical trials testing immune checkpoint modulators in colorectal cancer (according to https://www.clinicaltrial.gov/, 19th of June 2015).

Target	Compound	NCT tracker	Phase	Tumor type	Status	Combination (target)
Antagonistic monoclonal antibodies						

PD-1	**AMP-224** *<Amplimmune*	NCT01352884	I	Advanced solid tumors or CTCL	Completed	
		NCT02298946	I	mCRC	Recruiting	+ Stereotactic body RT + Cyclophosphamide
	**PDR001** *<Novartis*	NCT02404441	I II	Advanced malignancies incl. only PD-L1^+^ MSI-H CRC	Recruiting	
	**Nivolumab** BMS-936558 MDX-2206 ONO-4538 *<Bristol-Myers Squibb*	NCT01629758	I	Locally advanced or metastatic solid tumors	Completed	+ Denenicokin (IL-21)
		NCT02060188	II	Recurrent and mCRC: MSI-H and MSI-L	Recruiting	+ Ipilimumab (CTLA-4)
		NCT02408861	I	HIV-associated solid tumors	Not yet recruiting	+ Ipilimumab (CTLA-4)
		NCT00836888	I	Advanced malignant solid tumors in Japan	Not yet recruiting	
		NCT01714739	I	Advanced solid tumors	Recruiting	+ Lirilumab (KIR)
	**Pembrolizumab** MK-3475 *<Merck*	NCT02054806	I	Biomarker-positive solid tumors	Recruiting	
		NCT02179918	I	Advanced solid tumors	Recruiting	+ PF-05082566 (CD137)
		NCT02332668	I/II	Advanced Melanoma; advanced relapsed PD-L1^+^ malignancies	Recruiting	
		NCT01876511	II	MSI-H (non)-CRC	Recruiting	
		NCT02460198	II	Previously treated locally advanced unresectable/MSI-H mCRC	Not yet recruiting	
	**REGN2810** *<Regeneron*	NCT02383212	I	Advanced malignancies	Recruiting	+ Hypofractionated RT + Cyclophosphamide
	**BGB-A317** *<BeiGene*	NCT02407990	I	Advanced cancers	Recruiting	
	**MEDI0680** AMP-514 *<AstraZeneca*	NCT02013804	I	Advanced cancers	Recruiting	
		NCT02118337	I	Advanced cancers	Recruiting	+ MEDI4736 (PD-L1)

PD-L1	**MEDI4736** *<AstraZeneca*	NCT01693562	I/II	Solid tumors	Recruiting	
		NCT02227667	II	Advanced CRC	Recruiting	
	**MDX-1105** BMS-936559 *<Bristol-Myers Squibb *	NCT00729664	I	Relapsed/refractory solid tumors (incl. CRC)	Active, not recruiting	
	**Avelumab** MSB0010718C *<MerckKGaA and Pfizer*	NCT01943461	I	Metastatic/locally advanced solid tumors	Recruiting	
		NCT01772004	I	Solid tumors	Recruiting	
	**MPD-L3280A** MSB0010718C *<Roche*	NCT01375842	I	Locally advanced/metastatic solid tumors incl. CRC	Recruiting	
		NCT01633970	I	Locally advanced or metastatic solid tumors (incl. >10 patients with CRC)	Recruiting	+ Avastin (VEGF) + Chemotherapy
		NCT02350673	I	Metastatic/locally advanced solid tumors	Not yet recruiting	+ RO6895882 (IL-2)
		NCT01988896	I	Metastatic/locally advanced solid tumors incl. KRAS-mutant mCRC	Not Yet recruiting	+ Cobimetinib (MEK)

CTLA-4	**Ipilimumab** MDX-010 YERVOY *<Bristol-Myers Squibb *	NCT01750983	I	Advanced or metastatic cancer	Recruiting	+ Lenalidomide
		NCT02239900	I/II	Advanced solid tumors with spread to liver, lung, or adrenal gland	Recruiting	+ Stereotactic body radiation
	**Tremelimumab** Ticilimumab CP-675,206 *<Pfizer*	NCT00313794	II	mCRC	Completed	
		NCT01975831	I	Advanced solid tumors (incl. CRC)	Recruiting	+ MEDI4736 (PD-L1)
		NCT02261220	I	Advanced solid tumors	Recruiting	+ MEDI4736 (PD-L1)

LAG-3	**BMS-986016** *<Bristol-Meyers Squibb*	NCT01968109	I	Solid tumors	Recruiting	+ Nivolumab (PD-1)
	**LAG-525** *<Novartis*	NCT02460224	I II	Advanced solid tumors (incl. PD-L1^+^CRC MSI-H)	Not yet recruiting	+ PDR001 (PD-1)

CD70	**ARGX-110** *<arGEN-x*	NCT01813539	ɪ	Refractory or relapsing CD70^+^ malignancies	Recruiting	

Agonistic monoclonal antibodies						

CD27	**Varlilumab** CDX-1127 *<Celldex*	NCT01460134	I	Solid tumors (incl. CRC)	Recruiting	
		NCT02335918	I/II	Advanced refractory solid tumors (incl. CRC)	Recruiting	+ Nivolumab (PD-1)

CD134 (OX40)	**MEDI6469** *<AgonOx*	NCT02318394	I	Recurrent or metastatic solid tumors	Recruiting	
		NCT02205333	I/II	Advanced solid tumors/aggressive B-cell lymphomas	Recruiting	+ Tremelimumab (CTLA-4) + MEDI4736 (PD-L1) + Rituximab
	**MEDI6383** *<AgonOx*	NCT02221960	I	Advanced solid tumors	Recruiting	
	**MOXR0916** RG7888 *<Genentech Inc.*	NCT02219724	I	Metastatic/locally advanced solid tumors	Recruiting	
		NCT02410512	I	Locally advanced, recurrent, or metastatic incurable solid tumors	Recruiting	+ MPL3280A (PD-L1)

Agonistic monoclonal antibodies						

GITR	**TRX518** *<Tolerx*	NCT01239134	I	Solid tumors/malignant melanoma	Recruiting	
	**MK-4166** *<Merck*	NCT02132754	I	Solid tumors	Recruiting	+ Pembrolizumab (PD-1)

CD137 (4-1BB)	**Urelumab** BMS-663513 *<Bristol-Meyers Squibb*	NCT01471210	I	Advanced solid tumors/B-cell NHL	Recruiting	
		NCT02110082	I	CRC/head and neck cancer	Recruiting	+ Cetuximab (EGFR)
		NCT02253992	I/II	Advanced solid tumor/advanced B-cell NHL	Recruiting	+ Nivolumab (PD-1)
	**PF-05082566** *<Pfizer*	NCT02444793	I/II	Advanced/metastatic solid tumors	Recruiting	+ Mogamulizumab (CCR4)

CD40	**CP-870,893** *<Pfizer*	NCT02225002	I	Advanced solid tumors	Completed	
		NCT00607048	I	Metastatic solid tumors	Completed	+ Paclitaxel/Carboplatin
	**RO7009789** *<Roche*	NCT02304393	I	Metastatic/locally advanced solid tumors	Recruiting	+ MPD-L3280A (PD-L1)
	**ADC-1013** *<AlligatorBioscience*	NCT02379741	I	Advanced solid tumors	Recruiting	
	**ChiLob 7/4** *<Southampton, UK*	NCT01561911	I	CD40^+^ solid tumors/refractory DLBCL	Completed	
	**SEA-CD40** *<Seattle genetics*	NCT02376699	I	Advanced metastatic tumors/unresectable solid malignancies	Recruiting	

PD-1, programmed death-1; PD-L1, programmed death ligand-1; CTLA-4, cytotoxic T lymphocyte antigen-4; CD, cluster of differentiation; LAG-3, lymphocyte activation gene-3; GITR, glucocorticoid-induced tumor necrosis factor receptor-related protein; CTCL, cutaneous T cell lymphoma; CRC, colorectal cancer; mCRC, metastatic CRC; MSI, microsatellite instability; MSI-H, MSI-high; MSI-L, MSI-low; HIV, human immunodeficiency virus; KRAS, Kirsten rat sarcoma viral oncogene; NHL, non-Hodgkin lymphoma; DLBL, diffuse large B cell lymphoma; IL, interleukin; KIR, killer-cell immunoglobulin-like receptor; RT, radiotherapy; VEGF, vascular endothelial growth factor; MEK, mitogen-activated protein kinase kinase; EGFR, epidermal growth factor receptor; CCR4, C-C chemokine receptor type 4.
